# Evaluating the Efficacy and Safety of Tirzepatide on Glycaemic and Non-glycaemic Outcomes in Diabetes: A Systematic Review of Meta-Analyses

**DOI:** 10.7759/cureus.56939

**Published:** 2024-03-26

**Authors:** Shilpa Kaore, Bhavya B, Sameer Khasbage, Shubham Atal

**Affiliations:** 1 Pharmacology, All India Institute of Medical Sciences, Bhopal, Bhopal, IND; 2 Pharmacology and Therapeutics, All India Institute of Medical Sciences, Bhopal, Bhopal, IND

**Keywords:** body weight, glycaemic control, incretins, dual agonist, type 2 diabetes mellitus, tirzepatide

## Abstract

Tirzepatide is a novel once-a-week dual glucose-dependent insulinotropic polypeptide (GIP) and glucagon-like peptide-1 (GLP-1) receptor agonist, recently approved for type 2 diabetes mellitus (T2DM) and obesity. A systematic review of the literature published in multiple meta-analyses on Tirzepatide with emphasis on its effect on glycaemic and non-glycaemic parameters was conducted. We systematically searched the electronic databases PubMed and Google Scholar up to August 2023 for meta-analyses that compared Tirzepatide with placebo or active antihyperglycaemic drugs in subjects with T2DM. Various parameters for efficacy and safety, with their point estimates and confidence intervals, such as glycated haemoglobin (HbA1c), fasting serum glucose (FSG), body weight, lipid, and cardiovascular outcomes were assessed. Six meta-analyses fulfilled the pre-specified criteria and were included in the study. In all the studies, Tirzepatide treatment at different doses resulted in a significant reduction in HbA1c and FSG levels along with a significant reduction in weight compared with active control and placebo groups. Tirzepatide significantly reduced levels of triglycerides and increased high-density lipoprotein (HDL) cholesterol, whether used as monotherapy or add-on therapy. The studies suggested the cardiovascular safety of Tirzepatide as there was no increase in major adverse cardiovascular events (MACE). The drug shows lesser hypoglycemia but predominant gastrointestinal adverse effects such as nausea, vomiting, and diarrhoea. In conclusion, Tirzepatide shows superior glycaemic control and weight loss in patients with T2DM with beneficial effects on lipids, without an increased risk of hypoglycemia and cardiovascular events.

## Introduction and background

Around the world, more than 422 million people live with diabetes mellitus (DM), the majority of whom reside in low- and middle-income nations. Diabetes is directly responsible for 1.5 million deaths annually. Over the past few decades, the disease has grown to pandemic proportions with a consistent rise in the overall prevalence and new cases of diabetes, particularly type 2 DM (T2DM) including among non-obese and young individuals [1]. Although numerous classes of drugs are available for T2DM with their own advantages, the drugs perceived as better are those that not only effectively lower blood glucose levels, but are also linked with a lower risk of hypoglycemia and weight gain, and have additional beneficial effects on cardiovascular or renal outcomes [2].

**Figure 1:**
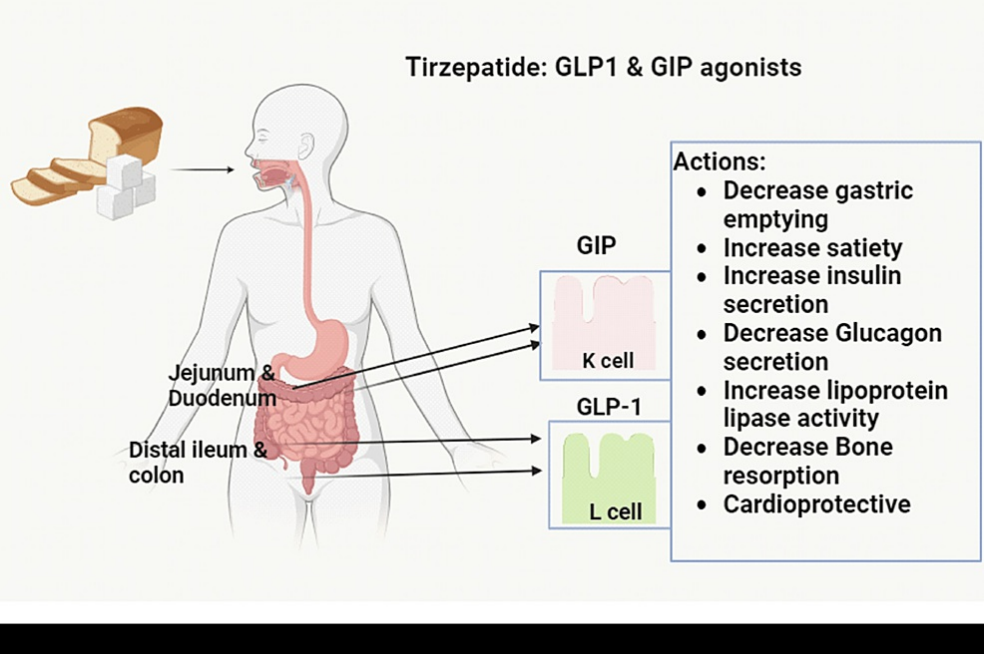
Main physiological actions of the dual GIP and GLP-1 agonist Created with BioRender.com by author BB. GIP: glucose-dependent insulinotropic polypeptide, GLP-1: glucagon-like peptide-1

Incretins are important substances released in the body physiologically to assist in maintaining normoglycaemia. The two main incretin hormones are glucose-dependent insulinotropic peptide (GIP) and glucagon-like peptide-1 (GLP-1) [3]. GIP is secreted by K cells in the duodenum and jejunum and GLP-1 is released from L cells in the distal ileum and colon; both contribute to the majority of the insulinotropic incretin actions [4]. GLP-1 agonists, which comprise short- and long-acting medications, have been linked to improved cardiovascular and glycaemic outcomes in patients with T2DM, and decrease in body weight in addition to having a high glycaemic efficacy [5]. A potentially ‘game-changing’ anti-diabetic drug named Tirzepatide has shown very promising results on glycaemia, body weight, as well as cardiovascular and renal parameters [6]. It works as an agonist for both GLP-1 and GIP receptors, giving it the cumulative effect of a stronger insulin response than is possible with these compounds individually (Figure 1) [2,3,7].

Multiple studies have been published focused on various outcomes of this drug in T2DM and obesity, for which it has received US Food and Drug Administration (FDA) approval [8,9]. A sizeable evidence base is available through meta-analyses of clinical trials exploring the effect of Tirzepatide on hyperglycaemia as well as other markers. There is a need to comprehensively combine the evidence and guide future research and prospective therapeutic applications, especially considering the progressively developing evidence. Hence, a systematic review of the literature published in the meta-analyses on Tirzepatide has been done, with specific emphasis on its effect on the different glycaemic and non-glycaemic parameters.

## Review

Methods

Search Strategy

A search was conducted systematically up to August 2023 in the two prominent and commonly used electronic databases - PubMed and Google Scholar for published studies, and MedRxiv and BioRxiv for pre-published studies. Additionally, we employed hand searching through manual searching of the bibliography of eligible articles, keywords and index terms such as MeSH terms for PubMed along with the Boolean operators AND/OR/NOT. For this purpose, a list of keywords to identify the eligible studies was prepared which included Tirzepatide, LY3298176, oral, dual agonist, twincretin, GLP-1, GIP, T2DM, T2DM, clinical, systematic review, meta-analysis.

Eligibility Criteria 

The inclusion criteria encompass any meta-analysis evaluating the safety, efficacy, and clinical pharmacology of oral Tirzepatide in T2DM. All published and pre-published studies meeting these criteria are eligible for inclusion, provided they are written in English and have full-text availability.

Conversely, exclusion criteria pertain to studies not conducted on human subjects, such as those involving animals, in-vitro experiments, or artificial intelligence analyses. Additionally, case reports, review articles, abstract-only publications, conference proceedings, editorial/commentary pieces, and book chapters are excluded. Studies with duplicate or overlapping data, as well as those with unreliable data extraction, are also excluded from consideration.

Data Collection

Primary screening was done by two teams of reviewers (SK, SNK and BB and SA) on the title and abstracts of the studies using the predefined study eligibility criteria. For this purpose, all the results found upon conducting the search were exported onto a Microsoft Excel sheet (Microsoft® Corp., Redmond, WA). All exported records (along with abstracts and weblinks added) were searched to remove duplicates using the “Author-Title-Year” criteria. All references that had the same title and author, and were published in the same year or the same journal were removed.

Secondary screening was further done on the full text of articles to confirm the eligibility of the primarily screened studies to assess their final suitability for inclusion. Relevant information and data were then extracted systematically from the texts of the eligible studies.

Data Extraction

Data was extracted from the included articles onto the Microsoft Excel sheet primarily by two of the authors (BB, SK) under the following headings: author(s), year of publication, journal, number of studies included, study design included, total population, interventional arms, mean age, mean duration of disease, period of intervention and follow up, mean change in HbA1c, fasting serum glucose (FSG) and body weight from baseline, incidence of adverse events (AEs)/serious adverse events (SAEs) and treatment discontinuation. Additionally, data elements like risk assessment, meta-analysis on HbA1c, FSG, weight and other outcomes (like lipid and cardiovascular) measured were also extracted. This was cross-checked by the two authors (SNK and SA), confirming all the extracted data after an independent review. In case of disagreement, a consensus was reached through discussion. The extracted data was then qualitatively analyzed thoroughly under different outcome categories.

Results

Study Characteristics

**Figure 2:**
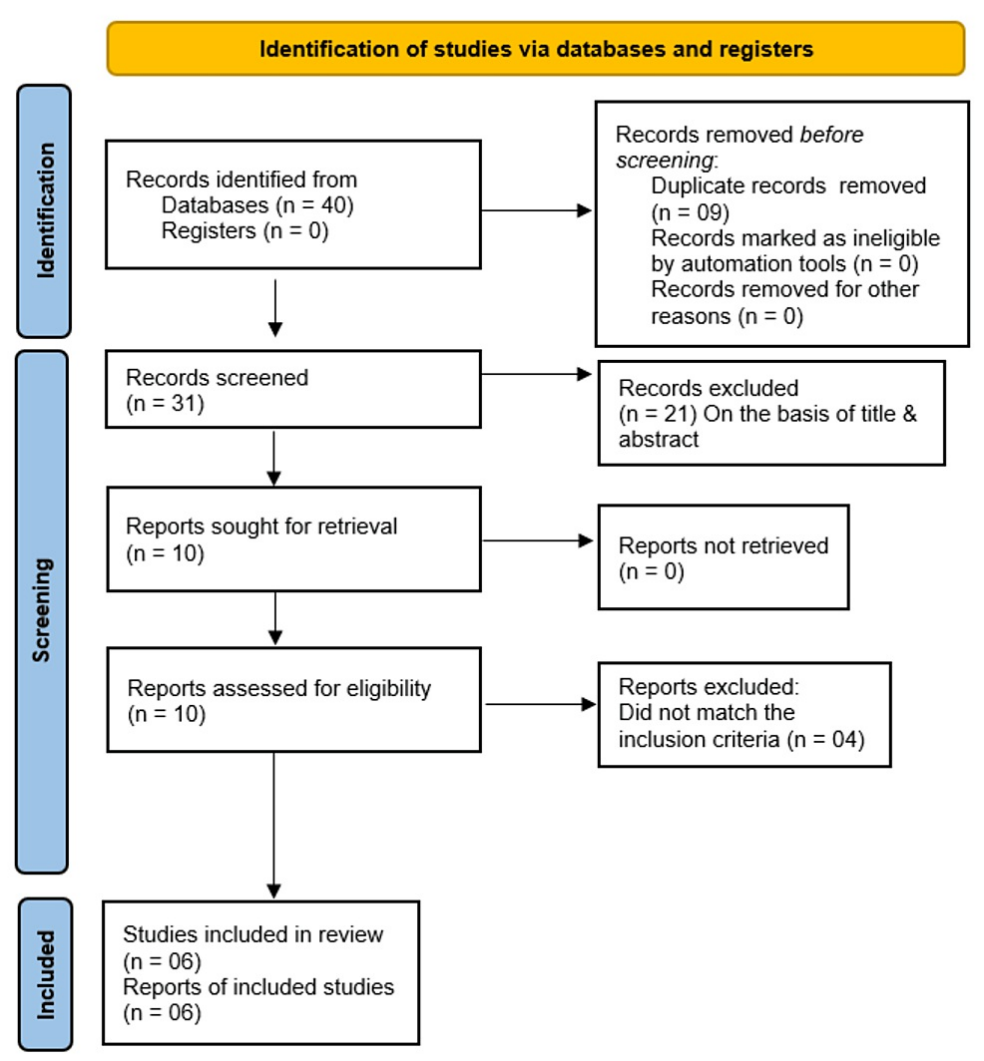
PRISMA flow diagram of the study PRISMA: Preferred Reporting Items for Systematic Reviews and Meta-Analyses

From the 40 articles identified after applying the search strategy, we excluded 34 articles, leaving six meta-analyses for assessment as is depicted in the Preferred Reporting Items for Systematic Reviews and Meta-Analyses (PRISMA) flow diagram in Figure 2. The detailed characteristics of the included studies are presented in Table 1 and key participant (patient) characteristics in Table 2. It is to be noted that these meta-analyses cover a total of eight original articles which are shown in Table 3. There was a significant overlap of studies included in the six meta-analyses chosen for this systematic review - one of the studies was included in all six meta-analyses (Frias et al., 2018), five studies were included in five of the six meta-analyses (Rosenstock et al., 2021 (SURPASS-1), Frias et al., 2021 (SURPASS-2), Ludvik et al., 2021 (SURPASS-3), Del Prato et al., 2021 (SURPASS-4), Dahl et al., 2022 (SURPASS-5)), one (Frias et al., 2020) was included in four meta-analyses, and only one (Inagaki et al., 2022 (SURPASS J-mono)) was unique in any of the meta-analyses. The total number of unique participants cumulatively across all eight original studies is 7245 (as analysed in the meta-analyses by Guan et al.). Table 3 shows details of the original randomized controlled trials (RCTs) which were included in various meta-analyses.

**Table 1:** Study characteristics

Study author (Year)	No. of trials	Total no. of participants	Dose of Tirzepatide	Control	Duration of intervention (weeks)	Duration of illness (years)
Karagiannis T et al. (2022) [10]	7	6609	5,10,15 mg Weekly	Placebo	26-52	8-14
				Semaglutide 1 mg		
				Dulaglutide 1.5 mg		
				Insulin degludec		
				Insulin glargine		
Bhagavathula A et al. (2021) [11]	4	2783	5,10,15 mg Weekly	Placebo	12-40	4-10
				Semaglutide 1 mg		
				Dulaglutide 1.5 mg		
Guan R et al. (2022) [12]	8	7245	5,10,15 mg Weekly	Placebo	12-52	4-10
				Semaglutide 1 mg		
				Dulaglutide 1.5 mg		
				Insulin degludec		
				Insulin glargine		
Tang Y et al. (2022) [13]	6	6579	5,10,15 mg Weekly	Placebo	12-52	4-14
				Semaglutide 1 mg		
				Dulaglutide 1.5 mg		
				Insulin degludec		
				Insulin glargine		
Dutta D et al. (2022) [14]	6	3484	10,12,15 mg Weekly	Placebo	12-52	8-14
				Semaglutide 1 mg		
				Dulaglutide 1.5 mg		
Sattar N et al. (2022) [15]	7	7215	5,10,15 mg Weekly	Placebo	12-52	9.3
				Semaglutide 1 mg		
				Dulaglutide 1.5 mg		
				Insulin degludec		
				Insulin glargine		

**Table 2:** Participant characteristics in included meta-analyses SD: standard deviation

Study	Age mean ± SD (years)	Body weight at baseline mean ± SD (kgs)	HbA1c at baseline mean ± SD (%)
Karagiannis T et al. [10]	58	91.5	8.2
Bhagavathula A et al. [11]	56.4±1.82	-	8.2
Guan R et al. [12]	57.9	90.3	6.6
Tang Y et al. [13]	52.9-63.8	84.8-96.3	7.85-8.59
Dutta D et al. [14]	53.6±12.8	84.8±20.0	8.05±0.80
Sattar N et al. [15]	58.7±9.9	91.12±20.3	8.3

**Table 3:** Details of various RCTs included in meta-analyses RCT: randomized controlled trials, SURPASS: Study of Tirzepatide in Participants with T2DM Not Controlled with Diet and Exercise Alone, SGLT2: sodium-glucose co-transporter 2

Meta-analysis	Studies	Background glucose-lowering therapy	Interventions	No. of participants
Karagiannis T et al. [10]	Frias et al., 2018 [16]	Drug naive (9.8%) or metformin monotherapy (90.2%)	Tirzepatide 5 mg, Tirzepatide 10 mg, Tirzepatide 15 mg, Placebo, Dulaglutide 1.5 mg	264
	Frias et al., 2020 [6]	Drug naive (13.4%) or metformin monotherapy (86.6%)	Tirzepatide 15 mg, Placebo	82
	Rosenstock et al., 2021 (SURPASS-1) [17]	Drug naive (54%) or previous oral medication use (46%)	Tirzepatide 5 mg, Tirzepatide 10 mg, Tirzepatide 15 mg, Placebo	478
	Frias et al., 2021 (SURPASS-2) [18]	Metformin monotherapy (100%)	Tirzepatide 5 mg, Tirzepatide 10 mg, Tirzepatide 15 mg, Semaglutide 1 mg	1878
	Ludvik et al., 2021 (SURPASS-3) [19]	Metformin monotherapy (68%) or metformin plus SGLT2 inhibitor (32%)	Tirzepatide 5 mg, Tirzepatide 10 mg, Tirzepatide 15 mg, Insulin degludec	1437
	Del Prato et al., 2021 SURPASS-4 [20]	Monotherapy with or any combination of metformin (95%), sulfonylurea (54%) or SGLT2 inhibitor (25%)	Tirzepatide 5 mg, Tirzepatide 10 mg, Tirzepatide 15 mg, Insulin glargine	1995
	Dahl et al., 2022 (SURPASS-5) [21]	Insulin glargine monotherapy (17%) or in combination with metformin (83%)	Tirzepatide 5 mg Tirzepatide 10 mg Tirzepatide 15 mg Placebo	475
Bhagavathula A et al. [11]	Rosenstock et al., 2021 (SURPASS-1) [17]	Drug naive (54%) or previous oral medication use (46%)	Tirzepatide 5 mg, Tirzepatide 10 mg, Tirzepatide 15 mg, Placebo	478
	Frias et al., 2018 [16]	Drug naive (9.8%) or metformin monotherapy (90.2%)	Tirzepatide 5 mg, Tirzepatide 10 mg, Tirzepatide 15 mg, Placebo, Dulaglutide 1.5 mg	264
	Frias et al., 2020 [6]	Drug naive (13.4%) or metformin monotherapy (86.6%)	Tirzepatide 15 mg, Placebo	82
	Frias et al., 2021 (SURPASS-2) [18]	Metformin monotherapy (100%)	Tirzepatide 5 mg, Tirzepatide 10 mg, Tirzepatide 15 mg, Semaglutide 1 mg	1878
Guan R et al. [12]	Frias et al., 2018 [16]	Drug naive (9.8%) or metformin monotherapy (90.2%)	Tirzepatide 5 mg, Tirzepatide 10 mg, Tirzepatide 15 mg, Placebo, Dulaglutide 1.5 mg	264
	Frias et al., 2020 [6]	Drug naive (13.4%) or metformin monotherapy (86.6%)	Tirzepatide 15 mg, Placebo	82
	Rosenstock et al., 2021 (SURPASS-1) [17]	Drug naive (54%) or previous oral medication use (46%)	Tirzepatide 5 mg, Tirzepatide 10 mg, Tirzepatide 15 mg, Placebo	478
	Frias et al., 2021 (SURPASS-2) [18]	Metformin monotherapy (100%)	Tirzepatide 5 mg, Tirzepatide 10 mg, Tirzepatide 15 mg, Semaglutide 1 mg	1878
	Ludvik et al., 2021 (SURPASS-3) [19]	Metformin monotherapy (68%) or metformin plus SGLT2 inhibitor (32%)	Tirzepatide 5 mg, Tirzepatide 10 mg, Tirzepatide 15 mg, Insulin degludec	1437
	Del Prato et al., 2021 SURPASS-4 [20]	Monotherapy with or any combination of metformin (95%), sulfonylurea (54%) or SGLT2 inhibitor (25%)	Tirzepatide 5 mg, Tirzepatide 10 mg, Tirzepatide 15 mg, Insulin glargine	1995
	Dahl et al., 2022 (SURPASS-5) [21]	Insulin glargine monotherapy (17%) or in combination with metformin (83%)	Tirzepatide 5 mg Tirzepatide 10 mg Tirzepatide 15 mg Placebo	475
	Inagaki et al., (SURPASS J-mono) 2022 [22]	None	Tirzepatide 5mg, Tirzepatide 10mg, Tirzepatide 15 mg, Dulaglutide 0.75 mg	636
Tang Y et al. [13]	Frias et al., 2018 [16]	Drug naive (9.8%) or metformin monotherapy (90.2%)	Tirzepatide 5 mg, Tirzepatide 10 mg, Tirzepatide 15 mg, Placebo, Dulaglutide 1.5 mg	264
	Rosenstock et al., 2021 (SURPASS-1) [17]	Drug naive (54%) or previous oral medication use (46%)	Tirzepatide 5 mg, Tirzepatide 10 mg, Tirzepatide 15 mg, Placebo	478
	Frias et al., 2021 (SURPASS-2) [18]	Metformin monotherapy (100%)	Tirzepatide 5 mg, Tirzepatide 10 mg, Tirzepatide 15 mg, Semaglutide 1 mg	1878
	Ludvik et al., 2021 (SURPASS-3) [19]	Metformin monotherapy (68%) or metformin plus SGLT2 inhibitor (32%)	Tirzepatide 5 mg, Tirzepatide 10 mg, Tirzepatide 15 mg, Insulin degludec	1437
	Del Prato et al., 2021 SURPASS-4 [20]	Monotherapy with or any combination of metformin (95%), sulfonylurea (54%) or SGLT2 inhibitor (25%)	Tirzepatide 5 mg, Tirzepatide 10 mg, Tirzepatide 15 mg, Insulin glargine	1995
	Dahl et al., 2022 (SURPASS-5) [21]	Insulin glargine monotherapy (17%) or in combination with metformin (83%)	Tirzepatide 5 mg Tirzepatide 10 mg Tirzepatide 15 mg Placebo	475
Dutta D et al. [14]	Frias et al., 2018 [16]	Drug naive (9.8%) or metformin monotherapy (90.2%)	Tirzepatide 5 mg, Tirzepatide 10 mg, Tirzepatide 15 mg, Placebo, Dulaglutide 1.5 mg	264
	Frias et al., 2020 [6]	Drug naive (13.4%) or metformin monotherapy (86.6%)	Tirzepatide 15 mg, Placebo	82
	Rosenstock et al., 2021 (SURPASS-1) [17]	Drug naive (54%) or previous oral medication use (46%)	Tirzepatide 5 mg, Tirzepatide 10 mg, Tirzepatide 15 mg, Placebo	478
	Frias et al., 2021 (SURPASS-2) [18]	Metformin monotherapy (100%)	Tirzepatide 5 mg, Tirzepatide 10 mg, Tirzepatide 15 mg, Semaglutide 1 mg	1878
	Ludvik et al., 2021 (SURPASS-3) [19]	Metformin monotherapy (68%) or metformin plus SGLT2 inhibitor (32%)	Tirzepatide 5 mg, Tirzepatide 10 mg, Tirzepatide 15 mg, Insulin degludec	1437
	Del Prato et al., 2021 (SURPASS-4) [20]	Monotherapy with or any combination of metformin (95%), sulfonylurea (54%) or SGLT2 inhibitor (25%)	Tirzepatide 5 mg, Tirzepatide 10 mg, Tirzepatide 15 mg, Insulin glargine	1995
	Dahl et al., 2022 (SURPASS-5) [21]	Insulin glargine monotherapy (17%) or in combination with metformin (83%)	Tirzepatide 5 mg Tirzepatide 10 mg Tirzepatide 15 mg Placebo	475
Sattar N et al. (2022) [15]	Frias et al., 2018 [16]	Drug naive (9.8%) or metformin monotherapy (90.2%)	Tirzepatide 5 mg, Tirzepatide 10 mg, Tirzepatide 15 mg, Placebo, Dulaglutide 1.5 mg	264
	Rosenstock et al., 2021 (SURPASS-1) [17]	Drug naive (54%) or previous oral medication use (46%)	Tirzepatide 5 mg, Tirzepatide 10 mg, Tirzepatide 15 mg, Placebo	478
	Frias et al., 2021 (SURPASS-2) [18]	Metformin monotherapy (100%)	Tirzepatide 5 mg, Tirzepatide 10 mg, Tirzepatide 15 mg, Semaglutide 1 mg	1878
	Ludvik et al., 2021 (SURPASS-3) [19]	Metformin monotherapy (68%) or metformin plus SGLT2 inhibitor (32%)	Tirzepatide 5 mg, Tirzepatide 10 mg, Tirzepatide 15 mg, Insulin degludec	1437
	Del Prato et al., 2021 (SURPASS-4) [20]	Monotherapy with or any combination of metformin (95%), sulfonylurea (54%) or SGLT2 inhibitor (25%)	Tirzepatide 5 mg, Tirzepatide 10 mg, Tirzepatide 15 mg, Insulin glargine	1995
	Dahl et al., 2022 (SURPASS-5) [21]	Insulin glargine monotherapy (17%) or in combination with metformin (83%)	Tirzepatide 5 mg Tirzepatide 10 mg Tirzepatide 15 mg Placebo	475
	Inagaki et al., 2022 (SURPASS J-mono) [22]	None	Tirzepatide 5mg, Tirzepatide 10mg, Tirzepatide 15 mg, Dulaglutide 0.75 mg	636

Effect of Tirzepatide on Glycaemic Outcomes

Overall, all eight original RCTs included in the different meta-analyses (Frias et al., 2018; Frias et al., 2020; Frias et al., 2021; Rosenstock et al., 2021; Ludvik et al., 2021; Del Prato et al., 2021; Dahl et al., 2022; Inagaki et al., 2022) compared different doses of Tirzepatide with the active control/placebo and all showed a significant improvement in glycaemic outcomes, with maximum effects being seen with 15 mg of Tirzepatide.

HbA1c: Different meta-analyses have been conducted to analyse the glycaemic efficacy of Tirzepatide. To evaluate the efficacy and safety of Tirzepatide in T2DM, Karagiannis et al. [10] carried out a meta-analysis which showed that in comparison to the placebo, GLP1 receptor agonist (GLP1RA), and basal insulin, Tirzepatide demonstrated a dose-dependent superiority in glycaemic control assessed through HbA1c. The reductions in HbA1c levels for Tirzepatide 5 mg, 10 mg, and 15 mg were as follows: -17.71 mmol/mol (3.8%) [(-21.66 to -13.75), I^2^= 83%, P<0.01], -20.20 mmol/mol (4%) [(-22.90 to -17.51), I2 = 53%, P = 0.12], and -22.35 mmol/mol (4.2%) [(-26.09 to -18.62), I2 = 78%, P< 0.01)]. When compared to GLP1RA, Tirzepatide 15 mg showed a significant reduction in HbA1c levels -10.06 mmol/mol (3.1%) [(-17.48 to -2.63), I^2^ = 89%, P< 0.01]. In comparison with basal insulin, 5 mg of Tirzepatide significantly reduced HbA1c levels: -7.66mmol/l (2.9%) [(-9.91 to -5.40), I^2^ = 82%, P = 0.02].

A meta-analysis and systematic review of randomised phase II/III clinical trials on the safety and efficacy of Tirzepatide in patients with T2DM was carried out by Bhagavathula et al. [11]. The included trials showed that different doses of Tirzepatide added to metformin or used alone showed varying degrees of glycaemic efficacy on HbA1c. Overall, Tirzepatide caused an HbA1c decrease of -1.94% (95% CI: -2.02 to -1.87, P< 0.001; I^2^ = 39.3%), 5 mg caused reduction of -1.79% (95% CI: -1.92 to -1.66), 10 mg caused reduction of -1.95% (-2.09 to -1.81), and 15 mg caused reduction of -2.09% (-2.22 to -1.96), respectively.

When compared to insulin, all doses of Tirzepatide showed a substantial improvement in HbA1c levels, however, in comparison to GLP1-RA, 10 mg and 15 mg of Tirzepatide were more effective according to a Bayesian network analysis conducted by Guan et al. [12]. The ranking probabilities for the various interventions were estimated using SUCRA (surface under the cumulative ranking score). The greatest probability of being the most effective option for lowering HbA1c (93.5%) was linked to 15 mg of Tirzepatide, followed by its dose of 10 mg (80.9%), 5 mg (62.4%), GLP1-RA (36.4%), insulin (26.5%), and placebo (0.3%).

Tang et al. [13], in their systematic review and meta-analysis, found Tirzepatide to significantly reduce HbA1c [MD (mean difference), -1.07% (-1.44, -0.56), I^2^ = 98%; P< 0.00001] when compared to the control. Tirzepatide add-on therapy [MD, -0.90%, (-1.24, -0.56)] and monotherapy [MD, -1.98%; (-2.22, -1.74)] both significantly decreased HbA1c. Additionally, they evaluated the efficacy of Tirzepatide in comparison to GLP-1RA and found that it reduced HbA1c significantly [MD, -0.36%; (-0.57, -0.15), I^2^=91%, P = 0.0008]. Additionally, there was a greater proportion of patients in the Tirzepatide group who achieved the HbA1c target of <7.0% (62.6% vs. 41.2%; RR, 1.87; 95% CI: 1.51, 2.33) (I2 = 92%; P< 0.00001), ≤6.5% (56.2% vs. 27.6%; RR, 2.43; 95% CI: 1.81, 3.28) (I^2^ = 92%; P< 0.00001), or <5.7% (27.5% vs. 6.0%; RR, 5.85; 95% CI: 1.74, 19.65) (I^2^ = 97%; P< 0.00001) when compared to control.

Dutta et al. [14] analysed data from four studies to determine the effect of Tirzepatide on HbA1c in comparison to dulaglutide, semaglutide, insulin degludec, and insulin glargine. Tirzepatide significantly reduced HbA1c from baseline (MD: 0.75% (1.05 to 0.45; P<0.01; I^2^=100%)). It also significantly reduced HbA1c when compared with placebo [MD: 1.93% (1.95 to 1.90); P<0.01; I^2^: 0%]. Upon comparison of Tirzepatide-treated patients to active controls, a substantially increased odds of reaching HbA1c <7% [Odds ratio (OR) = 4.28 (95% CI: 2.01-9.11); P< 0.01; I^2^ = 91%] and <6.5% [OR = 4.39 (95% CI: 2.44-7.92); P< 0.01; I^2^ = 90%] were reported. Table 4 depicts the change in HbA1c in the various meta-analyses.

**Table 4:** Change in the levels of HbA1c from baseline *Statistically significant (P < 0.05). I^2^: level of heterogeneity, CI: confidence interval, GLP-1RA: glucagon-like peptide-1 receptor agonist, HbA1c: hemoglobin A1c

Study	Comparators	Mean difference	P value	95% CI	I^2^ (%)
Karagiannis T et al. [10]	5 mg Tirzepatide vs placebo	-17.71	< 0.01	(-21.66, -13.75)	83
	10 mg Tirzepatide vs placebo	-20.20	0.12	(-22.90, -17.51)	53
	15 mg Tirzepatide vs placebo	-22.35	< 0.01	(-26.09, -18.62)	78
	5 mg Tirzepatide vs GLP-1RA	-3.22	0.22	(-05.64, -0.80)	34
	10 mg Tirzepatide vs GLP-1RA	-7.11	0.09	(-11.09, -3.14)	66
	15 mg Tirzepatide vs GLP-1RA	-10.06	< 0.01	(-17.48, -2.63)	89
Bhagavathula A et al. [11]	Overall Tirzepatide vs placebo/Active control	-1.94	< 0.001	(-2.02, -1.87)	39.32
	5 mg Tirzepatide vs placebo/Active control	-1.79	0.31	(-1.92, -1.66)	15.66
	10 mg Tirzepatide vs placebo/Active control	-1.95	0.82	(-2.09, -1.81)	00
	15 mg Tirzepatide vs placebo/Active control	-2.09	0.29	(-2.02, -1.87)	19.06
Guan R et al. [12]	5 mg Tirzepatide vs placebo	-0.13		(-1.03, 0.78)	
	10 mg Tirzepatide vs placebo	0.11		(-0.78, 0.99)	
	15 mg Tirzepatide vs placebo	1.70		(0.93, 2.47)	
Tang Y et al. [13]	Tirzepatide vs Active control monotherapy	-1.98	< 0.0001	(-2.22, -1.74)	98
	Tirzepatide vs Active control add-on therapy	-0.90	< 0.0001	(-1.24, -0.56)	98
	Tirzepatide vs GLP1 RA	-0.36	0.0008	(-0.57, -0.15)	91
Dutta D et al. [14]	Tirzepatide vs Active control	-0.75	< 0.01	(-1.05, -1.90)	100
	Tirzepatide vs placebo	-1.93	< 0.01	(-1.95, -1.90)	00

FSG: Different dosages of Tirzepatide were observed to lower FSG in comparison to the control/placebo group.

Bhagavathula et al. [11] reported that for three different doses of Tirzepatide (5 mg, 10 mg, and 15 mg), a significant reduction in FSG versus placebo/controls was observed, with values of -54.7 mg/dL (95% CI: -62.0 to -47.4), -43.6 mg/dL (95% CI: -50.2 to -36.9), -52.3 mg/dL (95% CI: -66.7 to -37.9), and -61.1 mg/dL (95% CI: -73.4 to -48.8), respectively.

Similar findings were reported by Tang et al. [13], who found that whether Tirzepatide was used as add-on therapy [MD, -13.54 ml/dl; (95% CI: -22. 78, -4. 30)] or monotherapy [MD, -59.17 mg/dl; (95% CI: -67.95, -50.39)], there was a significantly greater reduction in FSG of 21.50 mg/dl (95% CI: -34.44, -8.56) (I^2^ = 98%; P< 0.00001) than that in the control. Tirzepatide markedly reduced FSG [(MD, -13.00 mg/dl; (95% CI: -18.90, -7.10)] also on comparison to GLP-1 RA.

The results from the analysis by Dutta et al. [14] also showed that Tirzepatide significantly reduced FSG in comparison to dulaglutide, semaglutide, degludec, and glargine [MD = 0.75 mmol/L (95%CI: 1.05 to 0.45); P< 0.01; I^2^ = 100%]. A significant reduction was also seen when compared with placebo [MD = 3.42 mmol/L (95% CI: 4.08 to 2.76)5P< 0.01; I^2^ = 98%].

In the Bayesian network meta-analysis done by Guan et al. [12], 15 mg of Tirzepatide appeared to be the most effective option for significantly lowering FSG (86.6%) according to SUCRA analysis. This was followed by 10 mg of Tirzepatide (71.6%), insulin (65.4%), 5 mg of Tirzepatide (51.1%), GLP1-RA (25.2%), and placebo (0.1%). Table 5 depicts the change in FSG in these studies.

**Table 5:** Mean change in fasting serum glucose *Statistically significant (P < 0.05). I^2^: level of heterogeneity, CI: confidence interval, GLP-1RA: glucagon-like peptide-1 receptor agonist, ACG: active control group, PCG: placebo control group

Study	Comparators	Mean difference	P value	95% CI	I^2^ (%)
Bhagavathula A et al. [11]	Overall Tirzepatide vs. placebo/control	-54.72	0.04	(-62.05, -47.39)	72.78
	5 mg Tirzepatide vs placebo/control	-43.60	0.04	(-50.26, -36.94)	-
	10 mg Tirzepatide vs placebo/control	-52.35	0.04	(-66.73, -37.96)	75.94
	15 mg Tirzepatide vs placebo/control	-61.14	0.04	(-73.46, -48.82)	71.53
Guan R et al. [12]	5 mg Tirzepatide vs. placebo	-1.16		(-2.99, 0.66)	
	10 mg Tirzepatide vs. placebo	-0.17		(-1.97, 1.63)	
	15 mg Tirzepatide vs placebo	3.20		(1.63, 4.77)	
	5 mg Tirzepatide vs GLP-1RA	-1.10		(-0.46, 2.66)	
	10 mg Tirzepatide vs GLP-1RA	2.10		(-3.68, -0.52)	
	15 mg Tirzepatide vs GLP-1RA	2.20		(0.64, 3.76)	
Tang Y et al. [13]	Tirzepatide vs control	-21.50	< 0.0001	(-34.44, -8.56)	98
	Tirzepatide vs GLP 1RA	-13.00	< 0.0001	(-18.90, -7.10)	98
Dutta D et al. [14]	Tirzepatide vs ACG	-0.75	< 0.01	(-1.05, -0.45)	100
	Tirzepatide vs PCG	-3.42	< 0.01	(-4.08, -2.76)	98

Post-prandial glucose (PPG): The effect of Tirzepatide on PPG in comparison to ACG (active control group) was determined by analysing data from three trials in the meta-analysis done by Dutta et al. [14], when compared to active controls (GLP1 RAs and long-acting insulin analogues), Tirzepatide significantly reduced PPG [MD = ‑0.87 mmol/L (95% CI: ‑1.12 to ‑0.61); P< 0.0; I^2^= 99%]. The effect of Tirzepatide on PPG in comparison to PCG (placebo control group) was determined by analysing data from a single trial and PPG was considerably lower in those getting Tirzepatide than in those receiving a placebo [MD = -3.36 mmol/L (95% CI: ‑3.50 to ‑3.22); P< 0.01]. These are the RCTs included in meta-analyses like Frias et al., 2018 [16]; Frias et al., 2020 [6]; Rosenstock et al., 2021 (SURPASS-1) [17]; Frias et al., 2021 (SURPASS-2) [18]; Ludvik et al., 2021 (SURPASS-3) [19]; Del Prato et al., 2021 SURPASS-4 [20]; Dahl et al., 2022 (SURPASS-5) [21] which shows change in glycaemic parameters.

Effect of Tirzepatide on Non-glycaemic Outcomes

Body weight: Different doses of Tirzepatide were observed to lower body weight in comparison to the control/placebo group in all the original eight RCTs which have been included in the various meta-analyses. Baseline body weight ranged between 80-94 kgs at baseline in all the meta-analyses included in our study. Tirzepatide 10 mg and 15 mg were observed to exhibit statistically significant weight loss in all the studies.

In the study done by Karagiannis et al. [10], Tirzepatide 5 mg (- 6.31 kg [95% CI 4.38, 8.25], I^2^ = 70%), 10 mg (- 8.43 kg [95% CI 6.77, 10.09], I^2^ = 68%), and 15 mg (- 9.36 kg [95% CI 6.20, 12.53], I^2^ = 91%), all showed dose-dependent decreases in body weight in comparison to placebo. More patients receiving any of the three Tirzepatide doses consistently experienced body weight reductions compared to placebo -1.68 kg (95% CI 0.84, 2.52 [I^2 ^= 0%]) with Tirzepatide 5 mg and -7.16 kg (95% CI 4.86, 9.46 [I^2^ = 72%]) with Tirzepatide 15 mg. Tirzepatide induced a greater reduction in body weight than GLP-1 RAs. Tirzepatide 5 mg, 10 mg, and 15 mg had the following ORs for achieving a weight loss of at least 5% (compared to GLP-1 RAs): 1.96 (95% CI: 1.01, 3.80 [I^2^ = 61%]), 4.79 (95% CI: 1.95, 11.73 [I^2^ = 74%]), and 4.57 (95% CI: 3.38, 6.18 [I^2^ = 0%]). In comparison to GLP-1 RAs, Tirzepatide at all doses was more effective in weight loss.

In another study done by Bhagavathula et al. [11], Tirzepatide significantly reduced body weight compared to the placebo/controls by -8.4 kg (95% CI: -9.6 to -7.2), from -7.0 kg (95% CI: -7.9 to -6.0) for Tirzepatide 5 mg to -8.6 kg (95% CI: -9.6 to -7.6) for Tirzepatide 10 mg and (-8.6 kg, 95% CI: -10.9 to -6.3) for 15 mg, respectively.

In the network meta-analyses done by Guan et al. [12], in comparison to insulin, all the doses of Tirzepatide demonstrated greater efficacy in regulating body weight. Tirzepatide 10 mg and 15 mg caused statistically significant decreases in body weight when compared to GLP1-RA. As per SURCA values as well, 15 mg Tirzepatide (99.7%) appeared to be the most effective intervention for body weight control.

Tang et al. [13] found similar results where a significant reduction in weight was seen from baseline when compared with active control and placebo. Whether Tirzepatide was used as add-on therapy or monotherapy (monotherapy vs. active control (GLP-1 RA): MD: -7.40 kg; 95% CI: -8.71 to -6.09; add-on therapy vs. control: MD: -8.11 kg; 95% CI: -11.96 to -4.25), it significantly reduced body weight when compared to the control group. Compared to GLP-1 RA, a more significant reduction in body weight (MD: -3.34 kg; 95% CI: -3.85 to -2.83) was seen.

The results were also supported by Dutta et al. [14], who found Tirzepatide to show significantly greater body weight lowering compared to dulaglutide/semaglutide/degludec/glargine [MD = ‑8.63 kg (95% CI: ‑12.89 to ‑4.36); P< 0.01; I^2^ =100%]. Table 6 shows the changes in body weight in the included meta-analyses.

**Table 6:** Mean change in body weight Values are expressed as mean±SD. *Statistically significant difference compared to baseline. I^2^ indicates the level of heterogeneity, GLP-1RA: glucagon-like peptide-1 receptor agonist, ACG: active control group, PCG: placebo control group

Study	Comparators	Mean difference	P value	95% CI	I^2^ (%)
Karagiannis T et al. [10]	5 mg Tirzepatide vs placebo	-6.31	0.04	(-8.25, -4.38)	70
	10 mg Tirzepatide vs placebo	-8.43	0.05	(-10.09, -6.77)	68
	15 mg Tirzepatide vs placebo	-9.36	< 0.01	(-12.53, -6.20)	91
	5 mg Tirzepatide vs GLP-1RA	-1.68	0.67	(-2.52, -0.84)	00
	10 mg Tirzepatide vs GLP-1RA	-4.78	0.12	(-6.57, -3.00)	59
	15 mg Tirzepatide vs GLP-1RA	-7.16	0.06	(-9.46, -4.86)	72
Bhagavathula A et al. [11]	Overall Tirzepatide vs placebo/control	-8.47	0.06	(-9.66, -7.27)	90.11
	5 mg Tirzepatide vs placebo/control	-7.00	0.05	(-7.98, -6.02)	-
	10 mg Tirzepatide vs placebo/control	-8.64	0.05	(-9.66, -7.61)	66.98
	15 mg Tirzepatide vs placebo/control	-8.47	0.00	(-8.67, -7.27)	93
Guan R et al. [12]	5 mg Tirzepatide vs placebo	-2.26		(-6.28, 1.76)	
	10 mg Tirzepatide vs placebo	-1.45		(-5.36, 2.46)	
	15 mg Tirzepatide vs placebo	-4.40		(-7.80, -1.00)	
	5 mg Tirzepatide vs GLP-1RA	-2.10		(-5.47, 1.27)	
	10 mg Tirzepatide vs GLP-1RA	-6.00		(-9.40, -2.60)	
	15 mg Tirzepatide vs GLP-1RA	-8.60		(-12.08, -5.12)	
Tang Y et al. [13]	Tirzepatide vs control monotherapy	-7.40	< 0.00001	(-8.71, -6.09)	-
	Tirzepatide vs control add-on therapy	-8.11	< 0.0001	(-11.96, -4.25)	99
	Tirzepatide vs GLP1 RA	-3.34	< 0.00001	(-3.85, -2.83)	27
Dutta D et al. [14]	Tirzepatide vs ACG	-8.63	< 0.00001	(-12.89, -4.36)	100
	Tirzepatide vs PCG	-6.84	< 0.00001	(-8.02, -5.65)	97

These are the RCTs included in meta-analyses like Frias et al., 2018 [16]; Frias et al., 2020 [6]; Rosenstock et al., 2021 (SURPASS-1) [17]; Frias et al., 2021 (SURPASS-2) [18]; Ludvik et al., 2021 (SURPASS-3) [19]; Del Prato et al., 2021 SURPASS-4 [20]; Dahl et al., 2022 (SURPASS-5) [21]; Inagaki et al., 2022 [22] which shows change in non-glycaemic parameters like body weight.

Lipid Parameters

In the meta-analysis done by Dutta et al. [14], data from two studies (Frias et al., 2018; Frias et al., 2021) [16, 17] were used to compare Tirzepatide to ACG in terms of its effect on triglycerides and LDL-C. In comparison to dulaglutide/semaglutide, patients receiving Tirzepatide did not show significantly different triglyceride levels [MD: ‑0.60 mmol/L (95% CI: ‑1.34 to 0.13); P = 0.11; I^2^ = 100%] and LDL‑C [MD = 0.10 mmol/L (95% CI: ‑0.08 to 0.28); P = 0.27; I^2^ = 98%]. However, a significantly higher HDL-C level was seen with Tirzepatide when compared with ACG [MD: 0.04mmol/l (95% CI: 0.04 to -0.04) P<0.01]. When compared to placebo, patients on Tirzepatide showed significantly reduced levels of triglycerides [MD = ‑1.83 mmol/L (95%CI: ‑1.93 to ‑1.73) and LDL-C [MD: ‑0.19 mmol/L (95% CI: ‑0.24 to ‑0.14); P< 0.01]. A similar result was shown by the meta-analysis done by Tang et al. whereby, four RCTs were included (Frias et al., 2018; Del Prato et al., 2021; Ludvik et al., 2021; Rosenstock et al., 2021) [16,20,19,17] and on comparison with the control group, there was a decrease in percentage change of total cholesterol and triglycerides while a significant increase in HDL cholesterol for Tirzepatide was seen, whether used as monotherapy or add-on therapy.

Cardiovascular Outcomes

Blood pressure: Tang et al. [13] analysed results from four studies (Frias et al., 2018; Del Prato et al., 2021; Ludvik et al., 2021; Dahl et al., 2022) [16,20,19,21], and found a significant reduction in systolic blood pressure (SBP) (-2.87 mmHg (-5.35 to -0.39), P<0.02) and diastolic blood pressure (DBP) (-1.73 mm Hg (-3.43 to -0.03), P<0.05) when Tirzepatide was administered as monotherapy. Also, a significant reduction in SBP (-5.24 mmHg (-6.96 to -3.53), I^2 =^ 67%, P<0.03) was seen when it was administered as add-on therapy.

Major Adverse Cardiac Events (MACE)

MACE 3: As per the meta-analysis conducted by Sattar et al. [15], the comparisons among the treatment groups (placebo, semaglutide 1 mg, dulaglutide 1.5 mg, insulin degludec, insulin glargine) for the MACE-3 outcomes showed a hazard ratio (HR) of 0.83 (95% CI, 0.58-1.18) for Tirzepatide for MACE-3 (cardiovascular or undetermined cause, myocardial infarction (MI) and stroke); HR = 0.78 (95% CI, 0.56-1.08) for composite outcome of MACE-3 or HHF; HR = 0.67 (95% CI, 0.26-1.70) for HHF (hospitalized due to heart failure) and an HR = 0.80 (95% CI, 0.51-1.25) for all-cause death.

MACE 4: Tang et al. [13] reported no differences between the Tirzepatide and control groups for MACE-4 outcomes defined as a composite of cardiovascular death, non-fatal MI, non-fatal stroke, hospitalization for unstable angina [3.63% vs. 5.63%; RR: 0.76; (95% CI: 0.53 to 1.09), P = 0.14]. In the meta-analysis by Dutta et al. [14], a similar finding was seen with no difference between the Tirzepatide and control groups [RR=0.83 (95% CI: 0.48 to 1.44), P = 0.5].

Sattar et al. [15] reported that Tirzepatide was not associated with increased risk of the MACE-4 outcome [HR = 0.80 (95% CI: 0.57-1.11)], cardiovascular death [HR = 0.90 (95% CI: 0.50-1.61)], MI [HR = 0.76 (95% CI, 0.45-1.28)], stroke [HR = 0.81 (95% CI, 0.39-1.68)] and hospitalization for unstable angina [HR = 0.46 (95% CI: 0.15-1.41)] when compared with the control groups (placebo, semaglutide 1 mg, dulaglutide 1.5 mg, insulin degludec, insulin glargine). An HR of 0.73 (95% CI, 0.51-1.05) (P = 0.089) was found for the time to first occurrence of established MACE-4 in trials comparing Tirzepatide to insulin glargine, insulin degludec, and placebo combined. The HR for the first instance of verified MACE-4 in the SURPASS-4 population (Tirzepatide versus insulin glargine in a high-risk group) was 0.74 (95% CI, 0.51-1.08)] (P = 0.123).

MACE 6: Sattar et al. [15] also reported an HR of 0.76 (95% CI, 0.49-1.17) in the post hoc analysis of the comparisons between the Tirzepatide and control groups (placebo, semaglutide 1 mg, dulaglutide 1.5 mg, insulin degludec, insulin glargine) for coronary revascularizations. This included 0.67 (95% CI, 0.40-1.13) for urgent revascularizations and 0.77 (95% CI, 0.37-1.61) for non-urgent revascularizations. The HR for MACE-6 (combination of coronary revascularizations or HHF with MACE-4) was 0.79 (95% CI: 0.58-1.06). Thus, according to the pre-specified cardiovascular meta-analysis by Sattar et al., overall Tirzepatide did not raise the risk of MACEs in T2DM individuals when compared to control groups.

These are the RCTs included in the meta-analysis, namely, Frias et al., 2018 [16]; Rosenstock et al., 2021 (SURPASS-1) [17]; Frias et al., 2021 (SURPASS-2) [18]; Ludvik et al., 2021 (SURPASS-3) [19]; Del Prato et al., 2021 SURPASS-4 [20]; Dahl et al., 2022 (SURPASS-5) [21]; Inagaki et al., 2022 (SURPASS J-mono) [22] which shows no increased risk of MACEs in patients with T2DM on Tirzepatide.

Safety Outcomes

Safety outcomes from the included meta-analyses were reviewed systematically to address parameters such as hypoglycaemia, gastrointestinal AEs, treatment discontinuation due to AEs, SAEs, and mortality. Overall, all eight original RCTs included in the different meta-analyses (Frias et al., 2018 [16]; Rosenstock et al., 2021 (SURPASS-1) [17]; Frias et al., 2021 (SURPASS-2) [18]; Ludvik et al., 2021 (SURPASS-3) [19]; Del Prato et al., 2021 SURPASS-4 [20]; Dahl et al., 2022 (SURPASS-5) [21]; Inagaki et al., 2022 (SURPASS J-mono) [22]) compared different doses of Tirzepatide with the active control/placebo and all showed significantly decreased AEs.

Guan R et al. [12] concluded from eight RCTs with 7245 participants that in the probability of safety concerns among the interventions compared, insulin ranked the safest, followed by placebo, Tirzepatide 5 mg, GLP-1 RA, and lastly, Tirzepatide 10 and 15 mg respectively. It was concluded by the SUCRA analysis that overall safety for gastrointestinal AEs (nausea, vomiting, diarrhoea) was in the ranking order of insulin, placebo, Tirzepatide; risk increases with increase in dose. Tirzepatide therapy, when compared with GLP RA-1 or placebo, overall did not lead to an increase in adverse drug events. Moreover, all AEs were mild to moderate in intensity. Predominantly, the participants experienced gastrointestinal side effects with Tirzepatide (more incidence with higher doses) - being similar to GLP-1 RAs, with a small number proportion experiencing hypoglycemia (3%), or AEs leading to discontinuation of therapy (7%).

In the meta-analysis performed by Karagiannis et al. on seven RCTs [10], the overall safety concerns point to hypoglycaemia and gastrointestinal adverse effects, with the limitation being that findings can be only generalised for obese and metformin-based background therapy. The odds for hypoglycaemia (OR ranging from 0.17 with Tirzepatide 5 mg to 0.25 with Tirzepatide 15 mg) were not increased by Tirzepatide. Hypoglycaemia incidence was less with 5 or 15 mg Tirzepatide versus basal insulin. Severe hypoglycaemia was reported in a few patients across the trial participants who required assistance: 10/4414 (0.22%) for Tirzepatide, 1 with semaglutide, and 11/1000 (1.1%) for insulin glargine. The odds of gastrointestinal adverse effects with Tirzepatide such as nausea [15 mg - (OR = 5.60 [95% CI 3.12 to 10.06], I^2^ = 0%)], vomiting [(15 mg - OR = 5.50 [95% CI 2.40 to 12.59], I^2^ = 0%, 10 mg - (OR = 2.98 [95% CI 1.13 to 7.80], I^2^ = 0%)] and diarrhoea (15 mg - (OR = 3.31 [95% CI 1.40 to 7.85], I^2^ = 52%) was increased; other GI adverse effects were similar in profile versus GLP-1 RA. This might have led to treatment discontinuation in the Tirzepatide arm as more participants discontinued while on 15 mg versus GLP-1 RA as compared to 5 or 10 mg, which was comparable.

Tang Y et al. [13] concluded (six RCTs with 6579 subjects) that the overall risk of hypoglycaemia was low with Tirzepatide compared with placebo or other active hypoglycaemic drugs. The findings did not show an increased risk of hypoglycaemia with Tirzepatide versus control as evaluated in three categories: blood glucose <70 mg/dl (25.70% vs. 45.62%; RR, 1.48; 95% CI: 0.80, 2.74), blood glucose <54 mg/dl (3.86% vs. 11.39%; RR, 0.54; 95% CI: 0.24, 1.22) and severe hypoglycaemia (0.18% vs. 0.51%; RR, 0.52; 95% CI: 0.21, 1.32). However, Tirzepatide exhibits an increased risk of gastrointestinal AEs (as supported by other systematic reviews and meta-analysis), observed mainly when it was given as add-on therapy, but not in terms of pancreatitis or cholelithiasis that amount to severe AEs.

Dutta et al. [14] also evaluated six RCTs (N = 3484 participants) and put forth that the relative risk of treatment-emergent adverse events (TEAEs) [risk ratio (RR) = 1.43 (95% CI: 1.14-1.80); P< 0.01; I^2^ = 40%] and severe AEs [RR = 1.00 (95% CI: 0.64-1.57); P = 1.00; I^2^ = 49%] was not different. Pooled data from two studies including 1026 participants indicated lower occurrence of hypoglycaemia [RR= 0.32 (95% CI: 0.17-0.60); P< 0.01; I^2^ = 78%] as compared to insulin, GLP-1 RA; while in three studies with 393 participants, Tirzepatide showed higher incidence of hypoglycaemia [RR = 4.22 (95% CI: 1.26-14.15); P = 0.02; I^2^ = 0%] as compared to placebo [14]. Data from these three studies (Frias et al., 2018; Del Prato et al., 2021; Ludvik et al., 2021) [16,20,19] showed the TEAEs [risk ratio (RR) = 1.43 (95% CI: 1.14-1.80); P< 0.01; I^2^ = 40%] and SAEs [RR = 1.00 (95% CI: 0.64-1.57); P = 1.00; I^2^ = 49%] were not different in people on Tirzepatide compared to placebo. Moreover, patients receiving Tirzepatide had similar occurrence of the adverse effects as nausea [RR = 2.86 (95% CI: 0.56-14.52); P = 0.21; I^2^ = 97%; vomiting [RR = 2.63 (95% CI: 0.62-11.16); P = 0.19; I^2^ = 93%; and diarrhea [RR = 2.52 (95% CI: 0.92-6.92); P = 0.07; I^2^ = 93] as compared to active controls. Table 7 shows the AEs in the included meta-analyses.

**Table 7:** Safety outcomes of Tirzepatide vs active control/placebo MD: mean difference, OR: odds ratio, RR: risk ratio, CI: confidence interval, I^2^: heterogeneity, GLP-1 RA: glucagon-like peptide-1 receptor agonist

Study	No. of trials	Comparators	GI adverse events (nausea, vomiting and diarrhoea)							Hypoglycaemia	
Guan R et al. [12]	08		[MD (95% CI]							-	
		Tirzepatide 5 mg vs Placebo	[-1.16 (-2.99, 0.66)]								
		Tirzepatide 5 mg vs GLP-1 RAs	[1.10 (-0.46, 2.66)]								
		Tirzepatide 5 mg vs insulin	[-0.22 (-1.10, 0.66)]								
		Tirzepatide 10 mg vs Placebo	[-0.17 (-1.97, 1.63)]								
		Tirzepatide 10 mg vs GLP-1 RAs	[2.20 (0.64, 3.76)]								
		Tirzepatide 10 mg vs insulin	[0.88 (-0.91, 2.66)]								
		Tirzepatide 15 mg vs Placebo	[3.20 (1.63, 4.77)]								
		Tirzepatide 15 mg vs GLP-1 RAs	[2.00 (0.40, 3.60)]								
		Tirzepatide 15 mg vs insulin	[0.68 (-1.14, 2.49)]								
Karagiannis T et al. [10]	07		Nausea			Vomiting	Diarrhoea			[0R (95% CI), I2]	
			[0R (95% CI), I2]								
		Tirzepatide 5 mg vs Placebo	[3.02 (1.56, 5.86),0]			[2.51 (0.95, 6.61),0]	[2.09 (0.77, 5.69),51]			[1.60 (0.59, 4.39), 43]	
		Tirzepatide 5 mg vs GLP-1 RAs	[0.91 (0.65, 1.26), 3]			[0.68 (0.42, 1.10),0]	[1.22 (0.85, 1.74),0]			-	
		Tirzepatide 5 mg vs Basal insulin	[6.18 (3.93, 9.73),0]			[3.72 (2.06, 6.72),0]	[3.52 (2.46, 5.05),0]			[0.17(0.06, 0.48), 94]	
		Tirzepatide 10 mg vs Placebo	[3.66 (1.91, 7.02),0]			[2.98 (1.13, 7.80),0]	[2.26 (0.91, 5.60),44]			[1.97(0.18, 5.68), 50]	
		Tirzepatide 10 mg vs GLP-1 RAs	[1.00 (0.69, 1.45),11]			[1.11 (0.72, 1.70),0]	[1.51 (1.07, 2.15),0]			-	
		Tirzepatide 10 mg vs Basal insulin	[10.93 (5.39, 22.15),54]			[6.34 (3.69, 10.89),0]	[5.23 (3.74, 7.33),0]			[0.22(0.15, 0.34), 72]	
		Tirzepatide 15 mg vs Placebo	[5.60 (3.12, 10.06),0]			[5.50 (2.40, 12.59),0]	[3.31 (1.40, 7.85),42]			[2.27(0.71, 7.31), 86]	
		Tirzepatide 15 mg vs GLP-1 RAs	[1.34 (0.99, 1.80),0]			[1.81 (0.65, 5.08),68]	[1.48 (0.84, 2.64),39]			-	
		Tirzepatide 15 mg vs Basal insulin	[13.60 (8.93, 20.72),0]			[6.66 (3.90, 11.37),0]	[5.59 (4.01, 7.79),0]			[0.25(0.14, 0.46), 86]	
Tang Y et al. [13]	06		Nausea	Vomiting				Diarrhoea		< 70 mg/dl	<54 mg/dl
			[RR (95% CI), I2]							[RR (95% CI), I2]	
		Tirzepatide vs Active control monotherapy	[2.35(1.10, 5.04)]		[2.22(0.51, 9.61)]				[1.58(0.80, 3.1)]	[6.97(0.95, 51.14)]	[0.11(0.00, 2.59)]
		Tirzepatide vs Active control add-on therapy	[3.57(1.20, 10.66),96]		[2.68(1.46, 4.90), 65]				[2.43(1.44, 4.09), 87]	[1.30(0.70, 2.42), 96]	[0.60(0.26, 1.37), 89]

Discussion

This is the first systematic review of meta-analyses done on the efficacy and safety of Tirzepatide on glycaemic and non-glycaemic outcomes in T2DM. However, it is to be emphasized that these review findings reflect the outcomes from a core set of studies that have been analyzed in the included meta-analyses for the various key outcomes like glycaemic control, body weight, lipid parameters, cardiovascular outcomes, safety, etc. Besides these core studies, some new has been published on Tirzepatide after this systematic review’s cut-off date, in diabetes and obesity, which have been discussed separately. More meta-analyses keep getting published as well, continuing the overlap of included studies and claiming updation - these have also been addressed at the end of the discussion.

Glucose-lowering is a cornerstone of treatment for T2DM [16]. Preclinical and molecular evidence shows that GIP agonism may increase meal-stimulated insulin secretion, increase the sensitivity of adipose tissue to insulin and improve GLP1-mediated central satiety. Acting as glucagon regulators, GIP/GLP1 dual agonists offer a greater weight reduction than GLP1-Ras; prolong GI transit, and decrease hunger. Hence, the molecule Tirzepatide, a "twincretin," which is a novel synthetic peptide of 39 amino acids was developed which also improves insulin resistance and β-cell function biomarkers [17].

The US FDA approved Tirzepatide once weekly subcutaneous injections on May 13, 2022, to “improve blood sugar control in adults with T2DM, as an addition to diet and exercise”. The dose is adjusted between 5 and 15 mg based on tolerability to achieve blood glucose targets. Tirzepatide can be used as monotherapy or as an add-on to other medications in T2DM patients [8]. Additionally, the FDA authorised Tirzepatide on November 8, 2023, for “managing chronic weight in patients who are obese (BMI ≥ 30 kg/m^2^) or overweight (BMI ≥ 27 kg/m^2^) and have at least one weight-related comorbid condition”. Here, it is intended to be used in conjunction with a reduced-calorie diet and increased physical activity [9]. Also, Tirzepatide has been included in the recent version of the widely accepted ADA (American Diabetes Association) guidelines for the management of T2DM, wherein it has been considered as having very high glucose lowering and weight loss efficacy [18].

Glycaemic Outcomes

The approval for diabetes was based on the randomised phase III clinical trials SURPASS 1-5 (Study of Tirzepatide in Participants with T2DM Not Controlled with Diet and Exercise Alone) and SURPASS J-mono, which showed glycaemic efficacy and safety of Tirzepatide when administered once weekly to patients with T2DM in comparison to placebo and other hypoglycaemic medications [2]. Up to 52% of T2DM subjects in the SURPASS-1 trial attained normoglycaemic levels (HbA1c < 5.7%; <39 mmol/l).

The meta-analyses done by various authors, including these eight RCTs, depicted similar results, wherein dose-dependent superiority in glycaemic efficacy was seen when 5 mg, 10 mg and 15 mg of Tirzepatide were compared with placebo or basal insulin or GLP-1 RAs (semaglutide, dulaglutide) when used as a monotherapy or an add on therapy. Significant reductions in HbA1c and FSG levels were observed with all three doses, and the glycaemic effect persisted from week 12 to week 40. Additionally, there was no correlation found between this beneficial glycaemic effect with a higher risk of hypoglycaemia.

According to Bhagavathula et al. [11], the highest dosage of Tirzepatide had the best effect on lowering HbA1c, and no changes were observed at weeks 26 or 40. The pooled efficacy of outcome data, with different kinds of comparators (placebo or GLP-1 RA) in the same analysis, was the limitation of this study. Karagiannis et al. [10] conducted independent analyses for different outcomes based on the kind of comparator (placebo, GLP-1 RAs, basal insulin) in order to generate meta-analysis estimates that are more meaningful and therapeutically relevant. The results were in good agreement with those obtained by Tang et al., Dutta et al., and Guan et al. Apart from this, Tang et al. [13] carried out a more thorough investigation using Tirzepatide as a monotherapy and as an add-on, along with a dose-response analysis, demonstrating the significant potential for glycaemic control. Dutta et al. [14] concentrated on an in-depth review of patients using 10 mg of Tirzepatide since it was found to be the most tolerable dosage throughout the trials. The inherent pharmacological characteristics of investigational medications at various doses may account for some of the reported differences in clinical outcomes. PPG was also seen to be significantly improved in this meta-analysis. Nonetheless, more research in this area is possible given the limited data on PPG. Also, it is unclear how Tirzepatide and higher dosages of GLP1-RA compare in terms of efficacy and safety, further highlighting the need for more extensive evidence generation.

Non-glycaemic Outcomes

Body weight: While reviewing the meta-analyses for effect on weight, the study by Karagiannis et al. [10] demonstrated a significant dose-dependent reduction in body weight with Tirzepatide when it was compared with the GLP-1R agonists. Similar results were shown in the meta-analysis done by Tang et al. and Dutta et al., whereby Tirzepatide reduced body weight more than semaglutide 1 mg and dulaglutide 1.5 mg. It also resulted in a higher proportion of body weight reduction of ≥5%, ≥10%, and ≥15%. Also, in the meta-analysis done by Guan et al., higher doses (10, 15 mg) of Tirzepatide showed robust effectiveness in reducing body weight compared with Insulin or GLP1-RA [13,14]. However, in the meta-analysis done by Bhagvathula et al. [11], 10 mg and 15 mg of Tirzepatide did not differ in the extent of body weight reduction which might be due to the pooled efficacy of outcome which wasn’t the case with other studies. Also, data wasn’t available from the SURMOUNT trials 3, 4 and 5 till then.

With 650 million cases worldwide, obesity is the most common chronic illness. Its many consequences, such as cardiovascular disease and T2DM, are important causes of morbidity and mortality worldwide. Initially Tirzepatide, after its approval in May 2022 for T2DM, was used as an off-label drug for obesity. However, on November 08, 2023, it was approved for the management of obesity and overweight people with certain conditions. The approval was based on the phase 3 SURMOUNT-1 (Study of Tirzepatide in Participants with Obesity or Overweight) and SURMOUNT-2 trials. Adults with obesity in the SURMOUNT-1 trial experienced average weight reductions of 19.5% for the 10 mg dose and 20.9% for the 15 mg dose by week 72. Patients receiving a placebo, on the other hand, lost only 3.1% of their body weight. This was likely due to the additive benefit of targeting various endogenous nutrient-stimulated hormone pathways that have been linked to energy homeostasis, as Tirzepatide is both a GLP-1 and GIP receptor agonist. Participants in SURMOUNT-2 had mean body weight reductions up to 14.7% by week 72, 65% of patients receiving the 15 mg dose experienced reductions of 10% or more, and 31% experienced reductions of 20% or more [23].

Lipid and cardiovascular outcomes: As seen in the meta-analyses done by Tang et al. [13], and Dutta et al. [14], apart from a significant increase in blood HDL C levels with Tirzepatide, its effect on lipid parameters is similar to that observed with dulaglutide and semaglutide. A greater reduction in insulin resistance and glucagon levels was noted with Tirzepatide as compared to dulaglutide and semaglutide, which could potentially be a factor in improved glycaemic and metabolic results over GLP1 RAs. The findings of Tang et al. [13], additionally established that Tirzepatide can have a positive impact on the reduction of blood pressure. Cardiovascular safety is currently an important factor to be considered. The finding of non-difference in MACE-4 between Tirzepatide and control in the meta-analyses suggested cardiovascular safety of Tirzepatide. However, MACE significantly decreased when liraglutide, semaglutide, and dulaglutide were given to individuals with T2DM. Sattar et al. [15] also reported no increase in MACE 3 and MACE 6 along with its primary outcome, MACE 4. The ongoing SURPASS-CVOT phase 3 trial (NCT04255433) of 54 months period, which compares Tirzepatide with dulaglutide in patients with T2DM and high risk for MACE, could confirm the findings [24].

Safety: The overall safety assessment of Tirzepatide from the meta-analyses suggests a satisfactory profile with a lower occurrence of hypoglycaemia and predominance of gastrointestinal AEs - nausea, vomiting, and diarrhoea. Tirzepatide exhibits a similar occurrence of gastrointestinal AEs compared to the GLP-1 RAs while exhibiting more such events compared to the placebo group [12]. Few studies have suggested significantly lower GLP‑1R affinity of Tirzepatide as compared to the GLP‑1R analogues dulaglutide or semaglutide could possibly explain marginally lower gastrointestinal side effects with Tirzepatide, and further, the GIP agonism may contribute to the better gastrointestinal tolerability of Tirzepatide, while its translation into clinical evidence remains to be documented.

Karagiannis et al. [10] concluded that nausea was more frequent with Tirzepatide vs placebo, with 15 mg associated with a higher incidence of vomiting and diarrhoea; but odds of other gastrointestinal events were similar between Tirzepatide and GLP-1 RAs, except for diarrhoea with Tirzepatide 10 mg. A higher rate of discontinuation was observed with Tirzepatide 15 mg, while all Tirzepatide doses were safe in terms of SAEs and mortality. Owing to the well-established and superior beneficial effects of GLP-1 RA and Tirzepatide (even with the lowest 5 mg dose) on the glycaemic parameters as well as on weight reduction, the safety outcome may also be considered in view of the neutral effect of the placebo either on glycaemic parameters or body weight. Bhagvathula et al. documented Tirzepatide to exhibit an acceptable safety profile for use in T2DM. Guan et al. inferred that no statistical difference exists between GLP1-RA and Tirzepatide for the safety concerns while insulin caused fewer gastrointestinal events; while another meta-analysis, by Tang et al., documented an increased risk of gastrointestinal AEs with Tirzepatide mainly as add-on therapy but not in terms of pancreatitis or cholelithiasis [11-13]. Dutta et al. [14] commented that a comparison of the pooled data over 12-52 weeks with GLP-1 RA or insulin concluded that the occurrence of TAEs but not SAEs was significantly higher in people receiving tirzepatide as compared to the active controls. Further, Tirzepatide had a significantly lower occurrence of hypoglycemia as compared to those receiving dulaglutide/semaglutide/degludec/glargine.

Further Studies and Meta-Analyses on Tirzepatide

The systematic review of meta-analyses done in our study includes studies up till August 2023. However, few studies have been published later which could be included in future studies. Results of SURPASS-6 have been published recently in November 2023, in which it was seen that at week 52, the mean change in HbA1c was -2.1% with Tirzepatide (pooled cohort) compared to -1.1% with insulin lispro. The treatment differences were statistically significant, with Tirzepatide causing less hypoglycemia and a greater decrease in body weight [25].

In a Bayesian network meta-analysis published by Ding Y et al., published in January 2024, showed that 5 mg, 10 mg, and 15 mg of Tirzepatide showed better efficacy in lowering HbA1c than 1 mg of subcutaneous Semaglutide, (-0.22 [-0.40, -0.03]%, -0.42 [-0.60, -0.24]%, and -0.53 [-0.71, -0.35]%, respectively) and body weight (-1.48 [-2.53, -0.43] kg, -4.00 [-5.05, 28.95] kg, and -5.71 [-6.73, -4.68] kg, respectively). The incidence of GI adverse effects was significantly increased by 5 mg, 10 mg, and 15 mg of Tirzepatide (48.32%, 30.96%, and 21.07%) and 0.5 mg and 1 mg of subcutaneous semaglutide (33.54% and 24.77%, respectively), according to the SUCRA value [26]. Similar results were seen in the meta-analyses done by Zhou et al., published in October 2023, showing no clinically significant increase in the incidence of hypoglycaemic episodes, major AEs, or all-cause fatal AEs. Participants treated with weekly Tirzepatide attained HbA1c and body weight target values much lower than any other comparator. On the other hand, gastrointestinal adverse effects and decreased appetite were reported more frequently with Tirzepatide as compared to placebo or controls [27].

Another, meta-regression analysis was done by Rohani et al., on the effect of Tirzepatide on body weight. In congruence with other studies, here also, the overall findings showed a significant reduction in body weight (-11.34 kg [-12.79 to -9.88] P<0.001), body mass index (-3.11 [-4.36 to -1.86] kg/m^2^, P<0.001), and waist circumference (-7.24 [-10.12 to -4.36] cm, P< 0.001) [28]. A meta-analysis was published on the safety of Tirzepatide by Zeng Q et al., in October 2023, whereby, Tirzepatide was not found to be significantly associated with an increased risk of pancreatitis when compared to all control groups consisting of basal insulin (glargine or degludec), selective GLP1-RA (dulaglutide or semaglutide once weekly), and placebo (RR 1.46, [95% CI] 0.59 to 3.61; I2 = 0.0%, p = 0.436). When compared to placebo or basal insulin, Tirzepatide was significantly related to gallbladder or biliary disease (RR 1.97, [95% CI] 1.14 to 3.42; I2 = 0.0%, p = 0.558). However, this association was not observed with the risk of cholelithiasis, cholecystitis, or biliary disorders [29].

Overall, these comprehensive findings unequivocally show the clinical utility of Tirzepatide which will help all diabetes stakeholders in their decision-making, particularly in comparison with other injectables such as GLP-1 RA (weekly) or insulin preparations (daily) in T2DM patients. Further assessment of long-term safety for Tirzepatide remains to be explored, especially cardiovascular and renal safety. There is no specific trial that is currently being conducted to evaluate the effect of Tirzepatide on chronic kidney disease (CKD) patients, however, Tirzepatide and insulin glargine were compared during a median follow-up of 85-104 weeks in participants with T2DM, BMI ≥25 kg/m^2^, and high cardiovascular risk or cardiovascular disease in a post hoc analysis of pre-specified renal outcomes for SURPASS 4. It was reported that the Tirzepatide group had significantly lower composite kidney endpoints, [renal failure, renal mortality, decline in eGFR ≥40% from baseline, and new-onset macroalbuminuria (UACR >300 mg/g, A3)], P < 0.05; [HR 0.59 (95% CI: 0.43-0.80)] [30]. With the impressive profile of Tirzepatide in terms of safety and efficacy, and positive outlook in the 1-year clinical use in T2DM, it may be one of the most significant additions to the armamentarium of T2DM, particularly those with severely uncontrolled glycemia and obesity. Ongoing trials can lead to further updates in the recommendations or guidelines in the long run. As of now, the drug has been approved in the USA, European Union, Canada, and Australia for T2DM. However, it awaits approval in Asian countries. Owing to current availability as an injectable drug only, patient acceptability over the other established oral antidiabetic drugs (OADs) would certainly be difficult, along with high cost and lack of approvals on a global scale.

Limitations

While analysing these results, it is important to consider certain limitations. The most significant limitation is perhaps the small number of original studies (eight RCTs) which have been included in the multiple meta-analyses evaluated in this review; a significant overlap in ensuing results. A variable degree of statistical heterogeneity was seen in the analysis with active comparators. This could be due to variations in baseline hypoglycaemic drugs between the trials or in the efficacy of the two GLP-1 RA comparators - semaglutide and dulaglutide. Furthermore, in each trial, the total risk of bias evaluation was based just on the primary outcome of HbA1c change. Heterogeneity may also be caused by variables including different settings of RCTs, population ethnic disparities, follow-up duration, outcome selections, definitions, and ascertainment. Lastly, the trial duration of studies was limited, and long-term data on Tirzepatide were deficient, and not enough to evaluate the hard endpoints, such as cardiovascular events and all-cause death.

## Conclusions

In conclusion, our systematic review of meta-analyses demonstrated robust overall effects of Tirzepatide on T2DM. Treatment with Tirzepatide in various doses shows beneficial effects on HbA1c, FSG, body weight, lipid markers and cardiovascular outcomes without increasing the risk of hypoglycaemia. Tirzepatide does lead to GI adverse effects but does not show significant risks in terms of MACE outcomes and serious adverse events. Additionally, there is a dose-response effect of Tirzepatide therapy on HbA1c control, body weight reduction and nausea and vomiting increase but not on hypoglycaemia, diarrhoea, and pancreatitis.
